# Differential Effects of Serum Heat Treatment on Chemotaxis and Phagocytosis by Human Neutrophils

**DOI:** 10.1371/journal.pone.0054735

**Published:** 2013-01-22

**Authors:** Alexander R. Mankovich, Cheng-Yuk Lee, Volkmar Heinrich

**Affiliations:** Department of Biomedical Engineering, University of California Davis, Davis, California, United States of America; University of California, San Diego, United States of America

## Abstract

Neutrophils, in cooperation with serum, are vital gatekeepers of a host’s microbiome and frontline defenders against invading microbes. Yet because human neutrophils are not amenable to many biological techniques, the mechanisms governing their immunological functions remain poorly understood. We here combine state-of-the-art single-cell experiments with flow cytometry to examine how temperature-dependent heat treatment of serum affects human neutrophil interactions with “target” particles of the fungal model zymosan. Assessing separately both the chemotactic as well as the phagocytic neutrophil responses to zymosan, we find that serum heat treatment modulates these responses in a differential manner. Whereas serum treatment at 52°C impairs almost all chemotactic activity and reduces cell-target adhesion, neutrophils still readily engulf target particles that are maneuvered into contact with the cell surface under the same conditions. Higher serum-treatment temperatures gradually suppress phagocytosis even after enforced cell-target contact. Using fluorescent staining, we correlate the observed cell behavior with the amounts of C3b and IgG deposited on the zymosan surface in sera treated at the respective temperatures. This comparison not only affirms the critical role of complement in chemotactic and adhesive neutrophil interactions with fungal surfaces, but also unmasks an important participation of IgGs in the phagocytosis of yeast-like fungal particles. In summary, this study presents new insight into fundamental immune mechanisms, including the chemotactic recruitment of immune cells, the adhesive capacity of cell-surface receptors, the role of IgGs in fungal recognition, and the opsonin-dependent phagocytosis morphology of human neutrophils. Moreover, we show how, by fine-tuning the heat treatment of serum, one can selectively study chemotaxis or phagocytosis under otherwise identical conditions. These results not only refine our understanding of a widely used laboratory method, they also establish a basis for new applications of this method.

## Introduction

Heat exposure of serum can inhibit some or all viral activity in the serum while leaving properties like the pH, antibody content, and ionic composition largely unchanged. Exploiting this effect, serum heat treatment is a common method to protect laboratory personnel against infectious agents like HIV, and also has many other applications in clinical immunology or cell-culture biology [1], [2]. For example, it can inactivate components of the complement system and, therefore, is often used in conjunction with immunoassays that otherwise would be compromised by the presence of active complement proteins, such as complement-fixation tests [3], [4] or enzyme-linked immunosorbent assays (ELISA) [5], [6].

A closely related advantage of the heat inactivation of complement is that it allows us to examine the role of the complement system in innate immune interactions between a host and pathogenic invaders. Previous studies have demonstrated a strong correlation between heat treatment of serum and a diminished aptitude of innate immune cells to perform phagocytosis of, or chemotaxis toward, various pathogenic targets. For example, a near-complete reduction of phagocytosis of *Pneumococcus* by rat, mouse, rabbit, and guinea pig granulocytes was found to result from heat treatment of autologous sera at 56°C for 5 minutes [7]. The cells’ diminished phagocytic potential was attributed to the loss of opsonic activity, and thus to the presence of heat-labile opsonins in untreated sera. Similarly, the inhibition of phagocytic interactions of human neutrophils with various strains of *Staphylococcus aureus* and *S. epidermitis* in heat-treated serum was attributed to a reduced opsonization of the bacteria with complement [8]. Phagocytosis of the fungus *Candida albicans* decreased as well, by about 50%, in serum that had been heated to 56°C for 30 minutes [9]. Moreover, serum heat treatment was also shown to compromise the chemotactic potential of neutrophils. For example, heat treatment of serum at 56°C for 30 minutes resulted in a 50% reduction of migration of human peripheral blood leukocyte in a Boyden chamber, which was attributed to the inhibition of casein- and C3a-dependent chemotactic pathways [10]. Similarly, both random migration as well as directed chemotaxis of human neutrophils were found to be significantly reduced after serum heat treatment at 56°C for up to 15 minutes [11]. However, a systematic assessment of the effects of temperature-dependent serum heat treatment on both the chemotactic as well as the phagocytic activity of the same type of human immune cell is missing.

In our own single-cell in-vitro studies of host-pathogen interactions we routinely supplement buffers with heat-treated, usually autologous serum [12], [13]. This is done primarily to maintain innate immune cells in a quiescent state prior to their first contact with the pathogenic targets of interest, which ensures that the cell response commences from a well-defined baseline [14]. A closer look at the effects of serum heat treatment recently revealed a surprisingly strong dependence of our experimental results on the exact temperature that the sera had been exposed to. Remarkably, heat treatment at different temperatures appeared to affect the chemotactic and phagocytic activity of immune cells in a differential manner. These findings have important implications for both basic immunological research as well as clinical applications.

We therefore conducted an in-depth study of the effects of temperature-dependent serum heat treatment on interactions of human neutrophils with opsonized zymosan, an insoluble fraction from yeast cell walls often used to mimic fungal infection [15], [16]. The cell-target interactions are examined on two complementary levels, i.e., by flow cytometry, and in single-live-cell/single-target experiments. This two-pronged approach combines the statistical power of high-throughput data acquisition with the exceptional detail afforded by high-resolution (both temporal and spatial) inspection of one-on-one encounters between cells and their targets. In the single-cell experiments, individual cells and target particles are maneuvered first into close proximity of each other by dual-micropipette manipulation. To assess pure chemotactic activity, a chosen pair of non-adherent cell and target are held at a well-defined, easily adjustable distance for the desired period of time. If required, this technique also allows us to gently wash away chemoattractants, and to relocate the target at will to opposite sides of the cell. Eventually, the target particle is brought into contact with the cell and released, which allows us to examine cell-target adhesion (and if needed, to compensate for its lack by enforcing prolonged contact) as well as subsequent phagocytosis on a single-cell basis.

The opsonized zymosan particles used as targets in this study are prepared by pre-incubation in autologous sera that had been heat treated at various temperatures. To assess the effect of this treatment, we perform four types of experiments and interrelate their results. First, we characterize the amounts of C3b and IgG on the surface of opsonized zymosan by fluorescent staining. Second, we examine the purely chemotactic response of individual human neutrophils to these particles. Third, we quantify the time course of single-cell phagocytosis of the particles by neutrophils. Fourth, we carry out cell-target recognition bulk assays by flow cytometry (FCM).

## Results

### Temperature-dependent Opsonization of Zymosan with C3b and IgG

We first addressed the question how serum heat treatment at various temperatures affects the deposition of the most common heat-labile (C3b) and heat-stable (IgG) opsonins onto the surface of zymosan particles. The particles were incubated in buffers containing 10% autologous serum that had been heat treated at 40, 44, 48, 52, and 56°C. Particles incubated in buffer containing 10% untreated serum (which had been kept at 37°C for the same time as used to prepare the heat-treated samples) served as a positive control. Our negative control consisted of plain particles, i.e., particles that were never exposed to serum.

**Figure 1:**
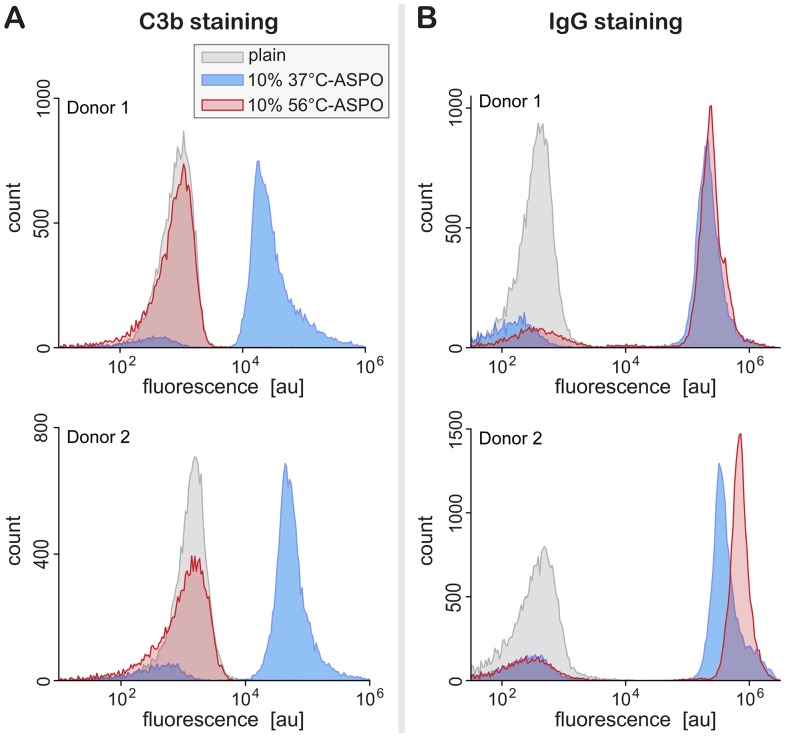
Example histograms of the fluorescence intensity of stained zymosan particles measured by flow cytometry. (**A**) Results of staining with FITC-conjugated anti-C3b. (**B**) Results of staining with Alexa-Fluor®-488-conjugated anti-IgG. In all cases, the particles had been incubated in three different buffers, i.e., serum-free HBSS (“plain”), HBSS with 10% untreated autologous serum (37°C-ASPO), and HBSS with 10% autologous serum that had been heat-treated at 56°C (56°C-ASPO). The legend included in (A) applies to all panels.

We fluorescently labeled the autologous-serum-pre-opsonized (ASPO) zymosan particles, as well as their plain counterparts, with FITC-conjugated anti-C3b or Alexa-Fluor®-488-conjugated anti-IgG antibodies. We then measured the fluorescence per particle of ∼20,000 particles of each sample by flow cytometry. Figure 1 presents example histograms of the fluorescence intensity of three different samples of zymosan particles. The fluorescence of plain particles (negative control) incubated with both staining antibodies was negligible. Large amounts of both complement C3b as well as immunoglobulins were detected on the surface of particles opsonized with normal serum (37°C; positive control). In contrast, we observed a clear difference between the efficiency of zymosan opsonization with C3b and IgG after incubation with 56°C-treated serum. Whereas the amount of complement fragments deposited by 56°C-treated serum onto the zymosan surface was diminished to the level of the negative control (Fig. 1A), the amount of detected IgG remained as high as the positive control at this temperature (Fig. 1B).

**Figure 2:**
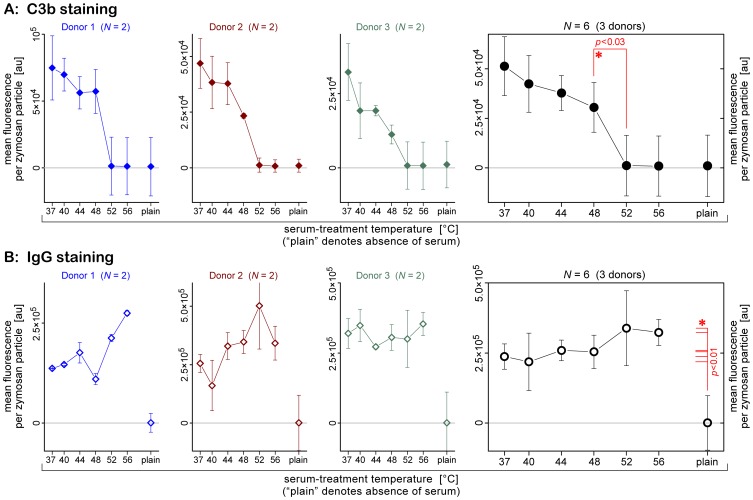
Summary of the FCM assessment of the temperature-dependent deposition of C3b and IgG onto the surface of serum-opsonized zymosan. (**A**) Results of staining with FITC-conjugated anti-C3b. (**B**) Results of staining with Alexa-Fluor®-488-conjugated anti-IgG. The first three columns depict example data obtained with sera from different, healthy donors. The rightmost column summarizes the results for a total of 3 donors. Values are block-centered means ± SD.

Figure 2 depicts the overall temperature dependence of the opsonization pattern of zymosan incubated with the heat-treated sera. A sharp decrease in C3b deposition occurred in the narrow range of serum-treatment temperatures of 48–52°C. Opsonization with serum that had been heated to 52°C or higher resulted in C3b levels on the zymosan surface that were as low as the negative control. In stark contrast, deposition of IgG onto the zymosan surface remained high at all serum-treatment temperatures. Importantly, the IgG levels stayed at all temperatures significantly above the negative control (with *p*-values ranging from <10^−4^ to 0.003), and did not consistently drop below the positive control.

### Temperature-dependent, Purely Chemotactic Activity in Single-cell/Single-target Encounters

We next explored how temperature-dependent serum heat treatment affects the aptitude of human neutrophils to sense a yeast-like fungal particle like zymosan from a distance. We use the term “pure chemotaxis” to denote a chemically triggered, local cell response that is not biased by interference from other, often simultaneously occurring processes, most notably adhesive cell-substrate interactions [13]. Usually a purely chemotactic response takes the form of a protrusion extended by the cell toward the chemotactic stimulus. It is best observed with cells that are not in contact with an adhesive substrate, such as cells that are held by gentle micropipette suction above the chamber bottom [13], [17], [18].

**Figure 3:**
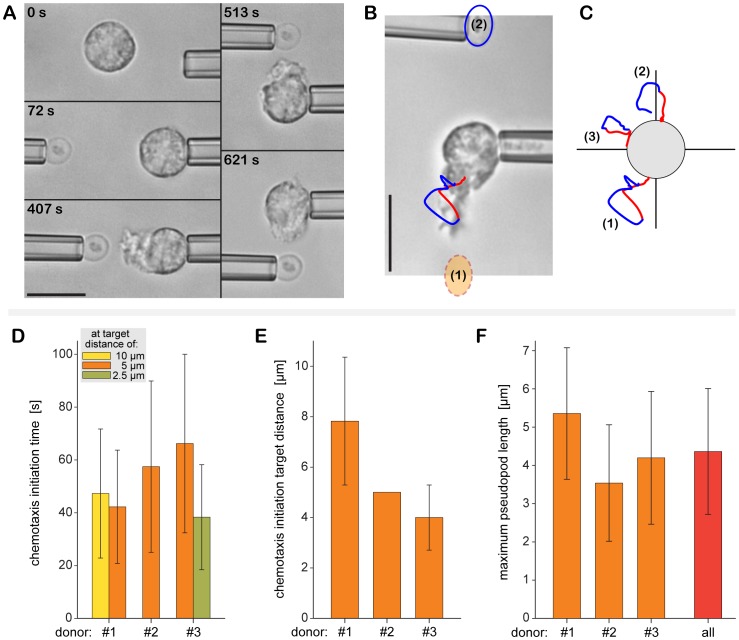
Single-cell/single-target experiment to quantify the purely chemotactic activity of non-adherent immune cells. (**A**) Using dual-micropipette manipulation, a zymosan particle is stepwise brought into close proximity of an initially passive neutrophil. In this configuration, chemotaxis takes the form of a cellular pseudopod that protrudes toward the zymosan particle and responds quickly to relocation of the particle. Relative times are included in each videomicrograph. (**B**) Analysis of the protrusion and retraction of pseudopods during complement-mediated chemotaxis of a neutrophil. The videomicrograph depicts the cell shortly after the zymosan particle had been relocated from position (1) to (2). Also shown is the positional trace of the tip of a pseudopod, where the time of particle relocation is indicated by a color change (*red → blue*). Thus the blue trace follows the (mainly retracting) pseudopod after removal of the local chemoattractant source from position (1). (**C**) Cumulative traces obtained sequentially for three target positions. (**D**) Time elapsed between the placement of target particles at the given distance and the initiation of a chemotactic pseudopod local to the target. (**E**) Average target distance at which pseudopod formation started within 3 minutes. (**F**) Maximum length of chemotactic pseudopods. The experiments analyzed in (D)-(F) were performed in buffer containing 48°C-treated serum. Scale bars in (A) and (B) denote 10 µm. Values in (D)–(F) are means ± SD.

Our approach to examine the purely chemotactic activity of non-adherent neutrophils is demonstrated in Fig. 3. In a typical experiment, a target particle and an initially quiescent cell were picked up at the tip of micropipettes and lifted above the chamber bottom. First, the cell and target were held at a distance of 10 µm for 3 minutes. If the cell responded by protruding a chemotactic pseudopod toward the target, the target was sequentially maneuvered to different locations around the cell (at well defined time intervals, and maintaining a consistent cell-target distance). On the other hand, if the cell did not exhibit chemotactic activity, the target was moved closer to the cell (to a distance of 5 µm, and eventually 2.5 µm), followed by the same procedure as above. Finally, the target was brought into soft physical contact with the cell and released from its pipette (see next section). Throughout the experiment, we continually adjusted the cell-aspiration pressure to maintain a more or less constant cell projection in the pipette (which provides an estimate of the cortical tension of the cell via Laplace’s law if needed [19]).

In buffer containing 10% autologous serum that had been heat treated at 48°C, twelve out of twelve neutrophils (from three donors) formed pronounced chemotactic pseudopods that clearly grew toward the respective positions of zymosan particles (Fig. 3A). Our analysis of this purely chemotactic behavior is illustrated in Figs. 3B and 3C. We tracked the tip of the protrusive pseudopod in recorded video images using custom-written software. Positional traces such as shown in Fig. 3C substantiated that the pseudopod did not gradually turn to follow the target particle to its new position. Instead, relocation of the zymosan particle led to the retraction of the previous pseudopod and the formation of a new one from an initially unperturbed cell-surface region facing the new particle position. The positional traces also allowed us to characterize the pseudopod’s time-dependent behavior, including its maximum extension as well as the speeds of protrusion and retraction.

Most examined cells sensed the particle over a distance of ∼5 µm and initiated the formation of a pseudopod within ∼1 minute after the zymosan particle was placed at this distance in buffer containing 48°C-treated serum (Figs. 3D and 3E). The average lengths of the pseudopods were in the range of ∼3–5 µm (Fig. 3F), and the average speeds of pseudopod extension and retraction were ∼27.0±7.2 (SD) nm/s and 32.2±7.6 (SD) nm/s, respectively. Intriguingly, there appeared to be consistent donor-dependent variations in chemotactic activity (Fig. 3), although a systematic exploration of this dependence will require a larger donor pool than used in this study. Similar cell behavior was observed with serum that had been treated at lower temperatures. In stark contrast, the neutrophil chemotactic activity was completely suppressed in serum that had been heated to 52°C (as verified with twelve cells from three donors) or higher.

### Quantitative Analysis of the Time Course of Temperature-dependent, Single-cell Phagocytosis

**Figure 4:**
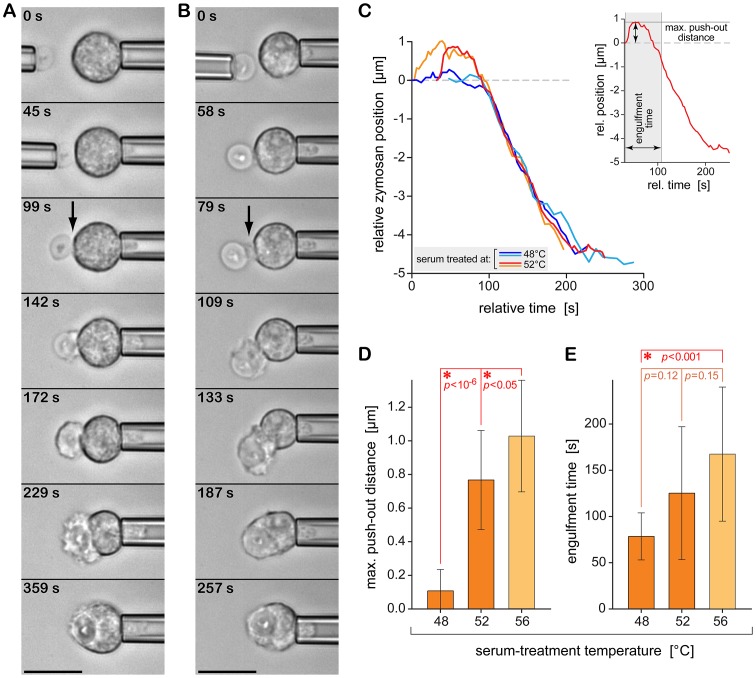
Single-cell/single-target experiment to quantify the time course of phagocytosis of initially quiescent immune cells. (**A**) Using dual-micropipette manipulation, a zymosan particle is “handed over” to an initially passive neutrophil. Subsequent videomicrographs illustrate the ensuing phagocytosis (relative times are included). An arrow marks the small pseudopodial “pedestal” that initially “pushes” the particle outwards. In this experiment, the zymosan particle was pre-opsonized with 48°C-treated serum and then washed. The experiment buffer was supplemented with 52°C-treated serum. (**B**) Same type of experiment as in (A), but here the particle was opsonized in the experiment buffer containing 52°C-treated serum. (**C**) Quantitative comparison of typical time-dependent positional traces of opsonized zymosan particles during single-cell phagocytosis by initially passive neutrophils. Two traces each are shown for zymosan opsonized with 48°C- and 52°C-treated serum, respectively. The individual time axes were aligned for maximum overlap during the inward movement of the particles. The inset illustrates our analysis in terms of the maximum push-out distance of the target particle and of the total engulfment time. (**D**) Average maximum push-out distances of zymosan from measurements such as illustrated in (C) for the two types of opsonized zymosan particles. (**E**) Average target engulfment times for the same experiments as analyzed in (D). Scale bars in (A) and (B) denote 10 µm. Also included in (D) and (E) are the results of previous measurements (shown in a lighter color) where zymosan had been opsonized in 56°C-treated serum. Values in (D) and (E) are means ± SD.

In previous work, we have extensively validated our dual-micropipette approach to quantify single-cell phagocytosis and demonstrated its ability to expose cell behavior that has eluded traditional techniques [12], [13], [14], [19], [20]. Here, the micropipette experiments were designed to inspect both chemotaxis (see previous section) as well as phagocytosis for each selected neutrophil (Fig. 4). Irrespective of whether or not a cell exhibited chemotactic activity, it was eventually maneuvered into physical contact with the respective target particle. The particle was then released from its holding pipette. Whether or not the cell was able to keep hold of the particle provided a qualitative measure of the overall strength of the cell’s adhesive interactions with the target surface. The cell behavior resulting from sustained cell-target contact allowed us to characterize the time course of the ensuing phagocytosis (Fig. 4).

Serum-treatment temperatures of up to (and including) 52°C did not impair neutrophil phagocytosis of zymosan particles in single-cell experiments that enforced physical contact between cell and target. Even though the neutrophils lacked chemotactic activity in interactions with zymosan particles opsonized with serum treated at 52°C (cf. previous section), the cells readily phagocytosed these particles. We note though that in some experiments, it was necessary to prolong the cell-target contact time in order to establish firm adhesion between the neutrophil and zymosan. In the presence of serum treated at 56°C, on the other hand, phagocytosis was somewhat reduced, although far from completely suppressed: 4 out of 35 neutrophils did not engulf opsonized zymosan particles even after prolonged cell-target contact. The neutrophil behavior in this situation was overall more irregular. Remarkably, cells from some donors appeared to exhibit a consistently stronger phagocytic capacity than others.

The staining experiments of Fig. 2 established that the opsonin composition on the zymosan surface changes drastically in the range of serum-treatment temperatures of 48–52°C. At the lower temperatures, the particles present a mixture of bound complement and antibodies, whereas at the higher temperatures, the prevalent opsonins are antibodies. The results of our single-cell experiments suggest that as long as the serum-treatment temperature remained below ∼56°C, this difference did not appear to affect the neutrophils’ principal aptitude to phagocytose zymosan particles. Yet this result still left room for the possibility that the detailed phagocytic cell response might be different for the two characteristic zymosan-opsonization patterns.

Hence, taking advantage of the ability of our single-cell approach to resolve small differences in the time course of phagocytosis, we next compared the time-dependent morphologies of neutrophils during the engulfment of zymosan particles presenting the different opsonization patterns. In particular, we examined the phagocytosis of zymosan particles opsonized in serum that had been treated at either 48°C or 52°C. Because we were only interested in phagocytic cell behavior here, the particles incubated with serum treated at 48°C were washed in serum-free buffer after pre-opsonization. For both target types, the buffer used in the actual single-cell experiments contained autologous serum treated at 52°C (which suppressed chemotaxis and helped to passivate the glass surfaces of cover slips and pipettes).

Careful inspection of the timelines of phagocytosis revealed a surprising difference between the neutrophil responses to zymosan particles whose surfaces presented these two distinct opsonization patterns (Fig. 4). In both cases, zymosan phagocytosis commenced with an initial protrusive cell deformation that at first “pushed” the attached particles outwards (i.e., away from the cell) by a small distance (marked by arrows in Figs. 4A and 4B). Accurate tracing of the particle position relative to the (opposite side of the) cell body allowed us to quantify the maximum outward target displacement (Figs. 4C and 4D). We found that this maximum push-out distance was significantly larger for zymosan opsonized with 52°C-treated serum [0.77±0.29 (SD) µm, *N* = 10] than in the case of zymosan pre-opsonized with 48°C-treated serum [0.11±0.13 (SD) µm, *N* = 16]. In strong correlation with this push-out distance, the total engulfment time (time elapsed between the first visible cell deformation local to the target and the closure of the phagocytic cup, Fig. 4E) was longer with 52°C-treated serum [it was 125±72 (SD) s for 52°C-treated serum versus 78±25 (SD) s for 48°C-treated serum]. Figures 4D and 4E also include data obtained in an earlier study where the same type of experiment was performed in buffer containing 10% autologous serum that had been heat treated at 56 (±2) °C (*N* = 22) [12]. Both the maximum push-out distance (Fig. 4D) as well as the target-engulfment time (Fig. 4E) are seen to exhibit a clear “dose response” in the temperature range from 48°C to 56°C.

### Temperature-dependent Target Recognition in Bulk

Our final set of experiments examined bulk interactions of human neutrophils with opsonized, FITC-labeled zymosan particles using flow cytometry. We again paid special attention to maintaining the quiescence of isolated neutrophils prior to their exposure to zymosan, and we minimized extraneous factors that might bias the outcome of such bulk measurements. For example, we avoided osmotic shock (such as inflicted when removing red blood cells by hypotonic lysis), as well as unnecessary compaction of cells by centrifugation, in particular in the presence of targets. Other measures included minimal fluid-shearing during blood draws and cell handling, and the use of calcium-free cell-storage buffers until incubation with the targets. Most importantly, we performed all recognition experiments with non-adherent neutrophils, keeping the cell-target mixtures under continual gentle agitation in suspension by placing them on a roller. This appeared to be the only way to ensure that the frequency of cell-target encounters was not affected by the serum preparation (as would be the case if cells and targets were in contact with a substrate because serum can strongly modulate the mobility of both cells as well as targets in that situation).

**Figure 5:**
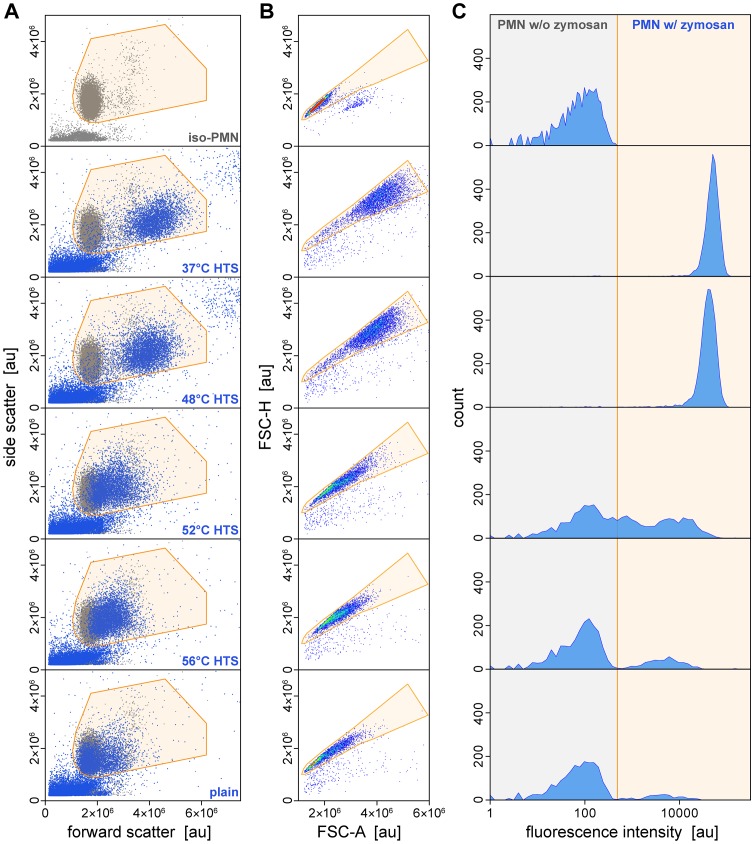
FCM analysis of bulk interactions of human neutrophils with zymosan particles in differentially heat-treated serum (HTS). (**A**) Plots of forward versus side scatter were used to delineate the region of FCM events that contains neutrophils. The scatter data of purified neutrophils (*top panel*) were included in all other panels for comparison (*gray symbols*). (**B**) Forward scatter height-versus-width density plots of the FCM events of the neutrophil gate defined in (A). These plots were used to further discriminate single-neutrophil events from coincidence events. (**C**) Histograms of the FITC fluorescence intensity of the events of the gate of (B) reveal varying degrees of neutrophil association with fluorescent zymosan particles. Also shown are the respective data for isolated neutrophils (obtained in the absence of zymosan; “iso-PMN”; *top row*), and for a mixture of neutrophils and un-opsonized zymosan (negative control; “plain”; *bottom row*).

**Figure 6:**
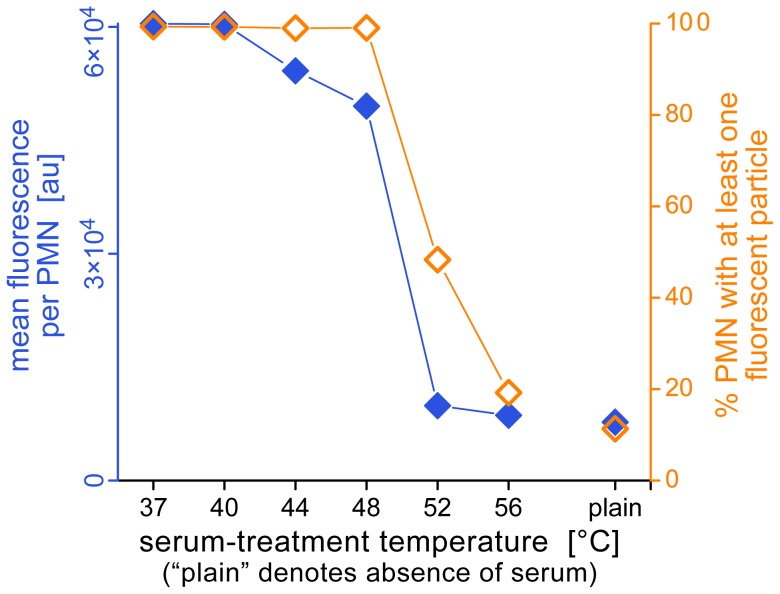
Example measurements of the uptake of fluorescent zymosan particles by human neutrophils. Fluorescence data such as shown in Fig. 5C were used to determine the mean fluorescence per neutrophil (this average includes all neutrophils), as well as the fraction of neutrophils that have associated with at least one zymosan particle, as a function of the serum-treatment temperature.

Figure 5 depicts a typical series of FCM measurements evaluating the effect of serum heat treatment at select temperatures. A set of neutrophil (PMN) aliquots were simultaneously incubated with zymosan in buffers containing differentially heat-treated, autologous serum. The graphs of Figs. 5A–5C not only illustrate the workflow of our analysis of the FCM data, but also present different views of the strong dependence of zymosan bulk recognition by neutrophils on the temperature used to treat the serum. Phagocytosis of opsonized zymosan was vigorous in sera that had been heated up to ∼48°C, but was strongly suppressed in sera treated at 52°C and 56°C. Our summary of this example series (Fig. 6) includes all used serum-treatment temperatures. Based on data such as shown in Fig. 5C, we employed two complementary measures to quantify the cell-target interactions: the mean fluorescence intensity per neutrophil, and the fraction of neutrophils associated with at least one zymosan particle [21].

**Figure 7:**
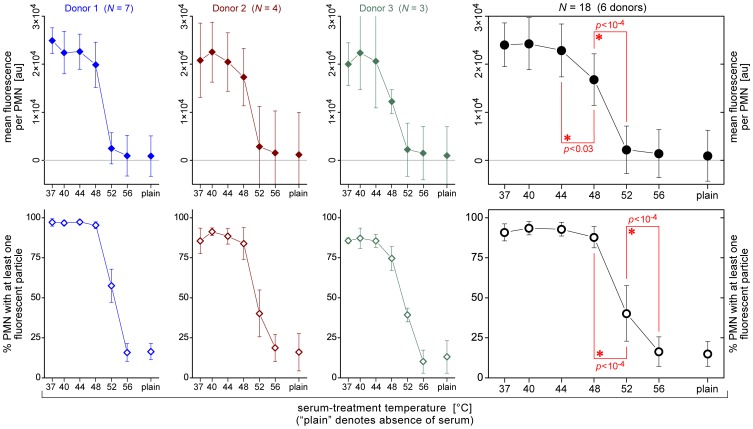
Summary of the FCM assessment of interactions between neutrophils (PMNs) and fluorescent zymosan particles in suspension as a function of serum heat treatment. The first three columns depict example data obtained with neutrophils and autologous sera from three different, healthy donors. The rightmost column summarizes the results for a total of 6 donors. Values are block-centered means ± SD.

Figure 7 presents example plots of the mean values (± standard deviations) of both of these measures for three donors, as well as a summary of all FCM phagocytosis experiments (consisting of 18 series of measurements for a total of 6 donors). The data corroborated the progressive inhibition of zymosan phagocytosis at moderate-to-high serum-treatment temperatures. Remarkably, the drop of the mean fluorescence per neutrophil to the level of the negative control occurred in a temperature range that was ∼3–4°C lower than the temperature window that marked the decrease of the fraction of neutrophils that had engulfed at least one particle (cf. also Fig. 6). For example, between 40% and 60% of neutrophils from our 3 main donors still recognized zymosan particles that had been pre-opsonized in buffer containing 52°C-treated serum (this fraction appeared to depend on the donor), but the mean fluorescence per cell was already quite low at this serum-treatment temperature (although often it was still higher than the fluorescence level of the negative control). It thus appeared that the temperature-dependent, overall phagocytic capacity of neutrophils (measured by the mean fluorescence per neutrophil) strongly correlated with serum-treatment conditions that resulted in zymosan opsonization with *both* C3b as well as IgG (cf. Fig. 2). On the other hand, the principal ability of neutrophils to recognize and phagocytose opsonized zymosan particles (measured by the fraction of neutrophils with at least one particle) appeared to mirror more closely the outcome of our single-cell phagocytosis experiments.

## Discussion

Although complement proteins are among the most-studied serum-borne effectors of the innate immune response, their actual contribution to the opsonic activity of serum often remains unclear. In addition, the role of complement is not limited to opsonization. For example, specific biochemical features on the surface of pathogens can activate complement enzymes, which then cleave other complement proteins and produce chemoattractant peptides [22], [23]. Facilitated by their small size, these peptides spread quickly by diffusion and recruit innate immune cells via chemotaxis. Such chemotactic priming also affects the adhesive and phagocytic potential of immune cells [22], providing an alternate route of phagocytosis enhancement by complement that is distinct from opsonization. In this study we have focused on two cornerstones of complement-mediated interactions between human neutrophils and pathogens. The first is opsonization of microbes with C3b, a step common to all three complement-activation pathways (i.e., the classical, alternative, and lectin pathways). The second is the production of chemoattractants at the surface of microbes, in particular the anaphylatoxin C5a, one of the most potent inflammatory peptides. C5a is produced downstream from C3b opsonization during complement interactions with microbes. Thus, heat inactivation of those complement enzymes that are required for opsonization with C3b (such as C3-convertases) is expected to impair C5a-mediated chemotaxis as well. However, it has been less clear whether chemotaxis-specific enzymes such as the C5-convertase remain active up to the temperatures that disable C3b opsonization.

The response of innate immune cells to serum-opsonized zymosan is broadly thought of as a prototypical example of complement-mediated host-pathogen interactions. At a first glance, this is a plausible assumption, given that zymosan originally was prepared to remove complement 3 from serum and thus has a high affinity to C3 [15], [24]. On the other hand, immunoglobulins constitute the second largest protein fraction (after albumin) of human serum, with IgGs being the most abundant sub-fraction (typical serum concentrations of IgG and C3 are 5–19 mg/mL and 0.8–1.5 mg/mL, respectively [25], [26]). The IgG fraction includes antibodies against the glycans β-glucan and mannan [27], [28], which together account for ∼75% of the dry mass of zymosan [16]. Therefore, it seems reasonable to expect that at least some antibodies participate in zymosan opsonization. We note that while we focus on the most common opsonins of fungal surfaces in this study, other serum components might also participate in zymosan opsonization.

Flow cytometry of fluorescently stained, opsonized zymosan particles confirmed that normal human serum deposits large amounts of both C3b as well as IgG onto this fungal model surface (Figs. 1 and 2). Moreover, an intriguing temperature dependence of the zymosan-opsonization pattern emerged when we labeled particles that had been incubated with differentially heat-treated serum. Whereas C3b deposition was progressively depressed at increasing serum-treatment temperatures, the amount of IgG on the zymosan surface remained more or less constant up to ∼48°C, and then appeared to increase slightly. Even though labeling of IgG with fluorescent secondary antibodies did not reveal a significant temperature dependence, we expect the microstructure of the deposited IgG to be affected by higher temperatures. Immunoglobulins are known to denature and aggregate at high temperatures [29], [30], consistent with the apparent slight increase in IgG-opsonization of zymosan. We also observed an increased forward-scatter in the FCM data at higher temperatures, indicating that the zymosan particles themselves were prone to clustering at these temperatures.

Our analysis of human neutrophil interactions with zymosan revealed that the temperature-dependent depression of chemotaxis and phagocytosis was not synchronized. For example, single-cell chemotactic activity appeared normal at 48°C but was completely eliminated in serum treated at 52°C. This chemotactic inactivation correlated well with our measurements of C3b levels on the zymosan surface, implying that pure neutrophil chemotaxis toward fungal targets was mediated by complement, presumably C5a [22]. Yet at the same serum-treatment temperature, all neutrophils in single-cell experiments, and ∼40% of cells in flow-cytometry bulk assays, were still able to phagocytose opsonized zymosan. Even when the serum had been heated to 56°C, the majority of neutrophils examined in single-cell experiments completed phagocytosis. We attribute this persistent recognition of zymosan by human neutrophils to IgG-mediated cell-target interactions, which also is supported by the presence of large amounts of IgG on the zymosan surface at all temperatures (bearing in mind though that IgG denaturation at high temperatures will eventually inhibit phagocytosis). Therefore, it seems reasonable to assume that IgG opsonization also plays an important role in the recognition of fungi opsonized with normal (untreated) serum. Our experiments have thus identified a narrow temperature window that can unmask the contribution of co-opsonization of fungal surfaces with IgG to the recognition of these surfaces by innate immune cells (a contribution that is obscured at lower serum-treatment temperatures by more prominent complement effects, and at higher temperatures by the inhibitory effect of heat-denatured IgG).

Why then is there such a noticeable difference (when using serum treated at temperatures ≥48°C) between the outcome of single-cell phagocytosis experiments and the overall phagocytic capacity reported by the mean fluorescence per neutrophil in bulk assays (top-right panel of Fig. 7)? The main difference between these two types of experiment lies in the nature of cell-target encounters. In bulk assays in suspension, contacts occur at random and, unless the cell is capable of holding on to the target, are very brief. In contrast, dual-micropipette manipulation allows us to control both the strength as well as the duration of enforced cell-target contacts. Our finding that these two types of cell-target contact lead to quantitatively different phagocytic behaviors evinces that the cells’ propensity to form sufficiently strong adhesive interactions with the zymosan surface is a key determinant of their overall phagocytic capacity, which also has been demonstrated by computer simulations of phagocytosis [20], [31]. In view of the low affinities of Fcγ receptors IIA and IIIB, a primary candidate that is likely to sustain cell-target adhesion is complement receptor 3 (CR3; synonyms: Mac-1, α_M_β_2_ integrin, CD11b/CD18) [32], [33]. CR3 is a highly regulated, multitasking integrin that is critically involved in leukocyte adhesion and migration [34], [35], [36]. One of CR3’s multiple ligands is C3b [37], [38]; however, to form a strong bond with C3b, CR3 must be in a high-affinity conformation [36], [39], [40]. The multitude of biochemical triggers that can activate CR3 include cross-linking of Fcγ receptors [41] and C5a-binding to the G-protein-coupled receptor C5aR (CD88). Our results show that neutrophil interactions with serum-opsonized zymosan stimulate both of these CR3-activating triggers, supporting the significance of CR3 as a key mediator of neutrophil adhesion to fungal surfaces [42]. Even at serum-treatment temperatures of 52°C or higher (where C3b is no longer deposited onto the zymosan surface), CR3 can still support adhesion via its binding region for β-glucans [43], [44], [45], presumably in cooperation with Fcγ receptors. Other receptors that can directly recognize native constituents of fungal surfaces do not appear to play a major role at this early stage of neutrophil-zymosan interactions. For example, human neutrophils do not express the mannose receptor [46] and only small amounts of β-glucan receptors like dectin-1 [33].

Intriguingly, the time-dependent neutrophil morphology during phagocytosis of zymosan changes significantly in the higher range of serum-treatment temperatures (Fig. 4). We have previously attributed the peculiar neutrophil “pedestal” that initially pushes adherent zymosan particles outwards to a weakening of cytoskeletal membrane anchors, i.e., structural linkages between the intracellular domains of engaged cell-surface receptors and the actin cytoskeleton [12], [13], [31]. This explanation is consistent with the progressive impairment of adhesive cell-target interactions at increasing serum-treatment temperatures. One might even speculate that the cell-morphology change could reflect a growing involvement of Fcγ receptor IIIB in cell-target attachments in this temperature range. FcγRIIIB is by far the most abundant Fc receptor on the surface of human neutrophils, and thus likely to encounter Fc domains, which densely coat the zymosan surface (Figs. 1 and 2). Moreover, the GPI-linked FcγRIIIB lacks an intracellular domain, consistent with our assertion that weaker cytoskeletal anchors lead to the formation of a neutrophil pedestal at the onset of phagocytosis of zymosan particles. Although this interpretation provides a plausible explanation of a variety of experimental and theoretical work, it remains speculative at this point. Relatively little is known about the function of FcγRIIIB (although there is recent support for the receptor’s involvement in outside-in signaling [47], [48]), and more work needs to be done to elucidate its possible role in the formation of the neutrophil pedestal at high serum-treatment temperatures.

### Conclusions

We confronted initially quiescent human neutrophils with zymosan particles that were incubated in buffers containing differentially heat-treated autologous serum. By combining single-cell chemotaxis and phagocytosis experiments with flow-cytometry recognition assays we were able to dissect and illuminate various mechanistic processes that contribute to the immune recognition of a range of opsonization patterns decorating the zymosan surface. This strategy has revealed a close correlation between complement (C3b) opsonization of zymosan and both the chemotactic activation as well as the adhesion-dependent phagocytic capacity of human neutrophils. On the other hand, we show that immunoglobulins also opsonize the zymosan surface in large numbers. Our results indicate that IgGs, in addition to facilitating the classical complement pathway, also participate more directly in the recognition of this fungal model surface. This participation appears to encompass not only a degree of purely antibody-mediated phagocytosis, but also the stimulation of FcγR-dependent signaling paths that lead to an enhancement of phagocytosis via the activation and/or upregulation of other receptors such as CR3. We show that neutrophils remain capable of engulfing opsonized zymosan even when the particles were incubated in serum treated at temperatures that disabled the complement system. In this case, cell-target adhesion could be mediated by CR3 binding of β-glucans and, possibly, an increased involvement of Fcγ receptors.

This study has shown how fine-tuned heat treatment of serum provides a simple means to selectively study chemotaxis or phagocytosis under otherwise identical conditions. Our results demonstrate the ability of this approach to not only deepen our understanding of a widely used laboratory method, but also expose new insight into fundamental immune mechanisms, including the chemotactic recruitment of immune cells, the adhesive strength of cell-surface receptors, the role of IgGs in fungal recognition, or the opsonin-dependent phagocytosis morphology of human neutrophils.

## Materials and Methods

### Ethics Statement

Written informed consent was obtained from all subjects (protocol approved by the Institutional Review Board, University of California Davis).

### Human Serum

Whole blood from healthy donors was drawn into silicone-coated collection tubes (Vacutainer; BD, Franklin Lakes, NJ, USA) and allowed to clot for 5 minutes. After centrifugation at 1200 g for 10 min at 20°C, the serum was filtered and deposited into 1.5 mL microcentrifuge tubes. The tubes were sequentially placed into an accurately temperature-regulated incubator (LE-509; MRC, Holon, Israel) and heat-treated for 45 min at the desired temperatures. Control samples and serum waiting to be treated were kept at 37°C for the same amount of time. The heat-treated serum was stored in 50 µL aliquots at -20°C for use within 4 weeks.

### Human Neutrophils

Neutrophils were isolated from heparinized blood of healthy donors as described previously [12], [19]. In short, blood was placed on top of 4 mL PMN separation medium (Matrix/Thermo Fisher Scientific, Waltham, MA, USA) and centrifuged at 700 g for 30 min. The recovered polymorphonuclear cell fraction was washed at 200 g for 10 min in Hanks’ balanced salt solution (HBSS, without calcium or magnesium; Sigma-Aldrich, St. Louis, MO, USA) containing 0.5% human serum albumin (HSA; Gemini, Sacramento, CA, USA). Finally, the cells were re-suspended in HBSS (without HSA) until use. About 90% of recovered polymorphonuclear cells were neutrophils (as assessed by optical microscopy).

### Zymosan Preparation and Temperature-dependent Opsonization

Non-fluorescent zymosan particles (Sigma-Aldrich) or FITC-labeled zymosan particles (Invitrogen, Carlsbad, CA, USA) were washed and suspended in phosphate buffered saline (PBS; USB Corp., Cleveland, OH, USA) at a concentration of 2 mg/mL, and then stored at 4°C. All serum-opsonized zymosan samples were prepared on the day of the experiment. The stock zymosan particles were washed twice and resuspended at a concentration of 0.12 mg/mL in HBSS (with calcium and magnesium) that contained 10% of the respective serum. This suspension was continually agitated on a rotator at room temperature for 1 h. Finally, the particles were washed twice more in the experiment buffer.

### Zymosan Staining

Opsonized and plain zymosan particles were incubated with 1 µg/mL FITC-conjugated mouse anti-human C3b (Accurate Chemical & Scientific Corp., Westbury, NY, USA) or Alexa-Fluor®-488-conjugated goat anti-human IgG (Invitrogen) monoclonal antibodies at room temperature on a rotator for 30 min, and washed twice. The mean fluorescence intensity per zymosan particle was measured using an Attune® Acoustic Focusing Flow Cytometer (Life Technologies, Carlsbad, CA, USA).

### Single-cell Experiments

Our micropipetting setup [49] and dual-micropipette phagocytosis experiments [12], [19] were described previously. In short, micropipettes with cylindrical, evenly broken tips of the desired inner diameter (typically 2–3 µm) were routinely manufactured before each experiment. Each pipette was mounted on a motorized 3-axis manipulator. Custom-written software allowed us to control up to two pipettes with a single game joystick. The shape of each pipette and the pipette-mounting angle enabled us to pick up cells and targets and lift them above the chamber bottom while providing a clear view of the aspirated cell projection.

The experiment chamber was created by trapping buffer solution (HBSS with calcium and magnesium, usually supplemented with 10% autologous serum) between two horizontal microscope coverslips. Apposing pipettes accessed the interior of this chamber through two open sides. Vertically movable water reservoirs controlled the pipette-aspiration pressure with high resolution. The aspiration pressure of the cell-holding pipette was monitored in real time by measuring the height difference between the reservoir connected to the pipette and a pre-zeroed reference reservoir.

For each experiment, a small volume of cell suspension (∼5 µL) was deposited into the chamber. To maintain the neutrophils in a quiescent state, the bottom coverslip had been coated covalently with 2-[methoxy (polyethyleneoxy) propyl] trimethoxysilane (PEG-silane; Gelest, Inc., Morrisville, PA, USA) as described earlier [14]. All single-cell experiments were carried out at room temperature.

### Bulk Cell-target Recognition Experiments in Suspension

Typically 250 µL of cells at 10^6^ cells/mL HBSS (with calcium and magnesium, usually supplemented with 10% of the respective autologous serum) were incubated with 30 µL of FITC-zymosan suspension at a cell:target ratio of ∼1∶10. The mixtures were placed on a rotator for 60 min (at room temperature), then fixed with 2% formaldehyde and diluted ∼20 times. The samples were analyzed using an Attune® Acoustic Focusing Flow Cytometer (Life Technologies).

### Statistical Analysis

Significance of the differences between means was established by ANOVA and Tukey’s test using Origin (OriginLab Corp.) or JMP (SAS Institute Inc.) software. Sets of flow-cytometry experiments that had been performed in parallel (e.g., when using the same batch of cells in a series of temperature-dependence tests) were analyzed as blocks; the means over repeats of such tests were obtained as block-centered means by ANOVA.
